# Hygroscopic Paper

**Published:** 1877-01

**Authors:** 


### HYGROSCOPIC PAPER.

The Journal of the Franklin Institute gives the following mode of preparing a useful hygroscopic paper: A bibulous paper is impregnated with a concentrated solution of chloride of cobalt. It is very sensitive to atmospheric variations, being blue in a dry atmosphere, changing to red when the air becomes humid. Four observations a day, made for a year, with every precaution, prove that this paper may be employed to indicate readily and precisely the hygrometric state of the air.

